# Association between genetic variants in the Coenzyme Q_10 _metabolism and Coenzyme Q_10 _status in humans

**DOI:** 10.1186/1756-0500-4-245

**Published:** 2011-07-21

**Authors:** Alexandra Fischer, Constance Schmelzer, Gerald Rimbach, Petra Niklowitz, Thomas Menke, Frank Döring

**Affiliations:** 1 Institute of Human Nutrition and Food Science, Devision of Molecular Prevention, Christian-Albrechts-University of Kiel, Heinrich-Hecht-Platz 10, 24118 Kiel, Germany; 2 Institute of Human Nutrition and Food Science, Devision of Food Science, Christian-Albrechts-University of Kiel, Heinrich-Hecht-Platz 10, 24118 Kiel, Germany; 3 Children's Hospital of Datteln, University of Witten/Herdecke, Dr.-Friedrich-Steiner Str. 5, 45711 Datteln, Germany

## Abstract

**Background:**

Coenzyme Q_10 _(CoQ_10_) is essential for mitochondrial energy production and serves as an antioxidants in extra mitochondrial membranes. The genetics of primary CoQ_10 _deficiency has been described in several studies, whereas the influence of common genetic variants on CoQ_10 _status is largely unknown. Here we tested for non-synonymous single-nucleotidepolymorphisms (SNP) in genes involved in the biosynthesis (CoQ3^G272S ^, CoQ6^M406V^, CoQ7^M103T^), reduction (NQO1^P187S^, NQO2^L47F^) and metabolism (apoE3/4) of CoQ_10 _and their association with CoQ_10 _status. For this purpose, CoQ_10 _serum levels of 54 healthy male volunteers were determined before (T_0_) and after a 14 days supplementation (T_14_) with 150 mg/d of the reduced form of CoQ_10_.

**Findings:**

At T_0_, the CoQ_10 _level of heterozygous NQO1^P187S ^carriers were significantly lower than homozygous S/S carriers (0.93 ± 0.25 μM versus 1.34 ± 0.42 μM, p = 0.044). For this polymorphism a structure homology-based method (PolyPhen) revealed a possibly damaging effect on NQO1 protein activity. Furthermore, CoQ_10 _plasma levels were significantly increased in apoE4/E4 genotype after supplementation in comparison to apoE2/E3 genotype (5.93 ± 0.151 μM versus 4.38 ± 0.792 μM, p = 0.034). Likewise heterozygous CoQ3^G272S ^carriers had higher CoQ_10 _plasma levels at T_14 _compared to G/G carriers but this difference did not reach significance (5.30 ± 0.96 μM versus 4.42 ± 1.67 μM, p = 0.082).

**Conclusions:**

In conclusion, our pilot study provides evidence that NQO1^P187S ^and apoE polymorphisms influence CoQ_10 _status in humans.

## Background

Coenzyme Q_10 _(CoQ_10_) is the predominant form of endogenous ubiquinone in humans. Synthesized in the mitochondrial inner membrane, CoQ_10 _is comprised of a ubiquinone head group attached to a trial of 10 five-carbon isoprenoid units, that anchors the molecule to the membranes [1]. Intracellular synthesis is the major source of CoQ_10_, however it can also be acquired through the diet and dietary supplements [2]. CoQ_10 _acts in the respiratory chain and is necessary for pyrimidine biosynthesis as well as a cofactor of uncoupling proteins [3]. CoQ_10 _has been also identified as a modulator of gene expression [4-6], inflammatory processes [7-9] and apoptosis [10,11].

The CoQ_10 _biosynthetic pathway comprises 10 steps, including methylations, decarboxylations, hydroxylations and isoprenoid synthesis and transfer [12]. The elucidation of this pathway was mainly due to studies in respiration-deficient mutans of *E. coli *and *S. cerevisiae *[13,14]. In humans, rare genetic variants in genes encoding enzymes of CoQ_10 _synthesis causes mitochondrial dysfunction, as CoQ_10 _carries electrons from complex I and complex II to complex III in the mitochondrial respiratory chain. Several forms of human CoQ_10 _deficiencies were characterized by infantile encephalomyopathy, renal failure, cerebellar ataxia or myopathy [15-17].

The complexity of CoQ_10 _biosynthesis suggests that genetic defects in different biosynthetic enzymes or regulatory proteins may cause different clinical syndromes. Although several studies have been undertaken to look into primary CoQ_10 _deficiency, the influence of common genetic variants on CoQ_10 _status is largely unknown. Therefore a proof of principle study in humans was performed to associate single nucleotide polymorphisms (SNPs) in genes encoding proteins of CoQ_10 _biosynthesis, reduction and metabolism with CoQ_10 _status before and after supplementation.

## Methods

### Participants and study design

Sample characteristics of subjects and study design have been recently described [18]. In short: 54 healthy male volunteers received 150 mg of the reduced form of CoQ_10 _(ubiquinol, KANEKA Corporation, Japan) daily in form of three capsules with each principal meal for 14 days. Fasting blood samples were taken before (T_0_) and after (T_14_) supplementation with ubiquinol from all study participants. The participants, aged 30.1 ± 6.7 years, had an average Body Mass Index (BMI) of 24.1 ± 2.5, no history of gastrointestinal, hepatic, cardiovascular or renal diseases, a habit of non- or occasional smoking (≤ 3 cigarettes/day) and maintenance of usual nutrition habits. The study was approved by the ethics committee of the Medical Faculty of Kiel University, Germany, and was conformed to Helsinki Declaration. All volunteers gave written informed consent.

### Genotyping

Genomic DNA was isolated from whole blood samples. Genotyping of all SNPs investigated (Table 1) was performed with the TaqMan system. Fluorescence was measured with ABI Prism 7900 HT sequence detection system (ABI, Foster City, USA).

**Table 1:** Selected polymorphisms in CoQ3, CoQ6, CoQ7, NQO1, NQO2 and apoE gene

Gene	refSNPid^a^	Sequence^b^	Position	Amino acid change
CoQ3	rs6925344	ACAATAC[C/T]TGCAATT	exon 6	Gly272Ser
CoQ6	rs8500	AGGTTCC[A/G]TGAGCCA	exon 11	Met406Val
CoQ7	rs11074359	ATGGTTA[T/C]GTTCAGG	exon 3	Met103Thr
NQO1	rs1800566	AGTTGAG[A/G]TTCTAAG*	exon 6	Pro187Ser
NQO2	rs1143684	CATGAAC[C/T]TTGAGCC	exon 3	Leu47Phe
apoE	rs429358	GGACGTG[C/T]GCGGCC	exon 4	Arg112Cys
apoE	rs7412	GCAGAAG[C/T]GCCTGG	exon 4	Arg158Cys

^a^: NCBI; ^b^: Applied Biosystems, *antisense

### HPLC analysis

CoQ_10 _analysis was based on the method of high-pressure liquid chromatography (HPLC) with electrochemical detection and internal standardisation using ubihydroquinone-9 and ubiquinone-9 as standards and has been described elsewhere [18].

### Statistical analysis

Data are expressed as means ± SD. Differences in the characteristics of the study population between two genotype groups were examined using the Student *t*-test and additionally for CoQ6^M406V ^the *χ*^2 ^-test in a dominant genetic model. To determine statistical significance between all genotypes, test for linear trend in one way analysis of variance (ANOVA) was performed. P-values ≤ 0.05 were considered statistically significant and all statistical analyses were computed using SPSS (Version 13.0). In order to analyze the impact of non-synonymous SNPs on the structure and function of proteins, PolyPhen server [19] was used. For power calculation, the GPower program (Version 3.1) was applied.

## Results and Discussion

### Selection of genes and single nucleotide polymorphisms

In order to identify common SNPs which may be associated with the CoQ_10 _status, we searched in the HapMap data base for non-synonymous variants in genes which are involved in CoQ_10 _biosynthesis and metabolism. As shown in table 1, we selected SNPs in the CoQ3 (rs6925344, C>T, Gly272Ser), CoQ6 (rs8500, A>G, Met406Val) and CoQ7 (rs11074359, T>C, Met103Thr) gene. These genes code for enzymes of CoQ_10 _biosynthesis. Functional variants [20,21] in the NQO1 (rs1800566, C>T, Pro187Ser) and NQO2 (rs1143684, T>C, Leu47Phe) gene were also included, as the encoded NAD(P)H:quinone oxidoreductases are involved in the recycling of CoQ_10_. Furthermore they protect cells from oxidative damage by catalyzing reduction of carcinogenic quinone compounds to their hydroquinone forms [22]. Two SNPs determining the apolipoprotein E (apoE) haplotypes E2, E3 and E4 (rs429358, rs7412) were further included. Both SNPs led to an amino acid change from cysteine to arginine at position 112 (rs429358) and 158 (rs7412), which gives rise to six possible diplotypes: E2/E2, E2/E3, E2/E4, E3/E3, E3/E4 and E4/E4. The apoE diplotypes have been associated with cholesterol metabolism [23,24], atherosclerosis [25], inflammation [26], lipid peroxidation [27] and longevity [28].

### Genotype distributions in the cohort

**Figure 1:**
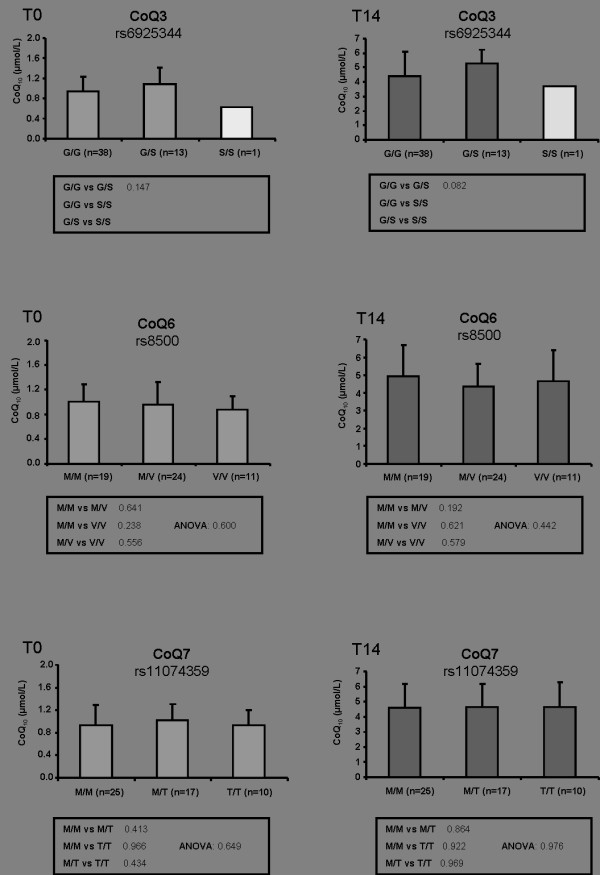
**Effect of amino acid exchange polymorphisms on CoQ_10 _plasma levels**. SNPs in genes encoding enzymes of the CoQ_10 _synthesis pathway (CoQ3^G272S^, CoQ6^M406V^, CoQ7M^103T^) before (T_0_) and after (T_14_) ubiquinol supplementation (150 mg/day) in humans are shown. Values are mean ± SD and n numbers (genotype distribution) are given in brackets. Differences between two genotype groups were examined using Student t-test and between all genotypes using "test for linear trend" (ANOVA).

**Figure 2:**
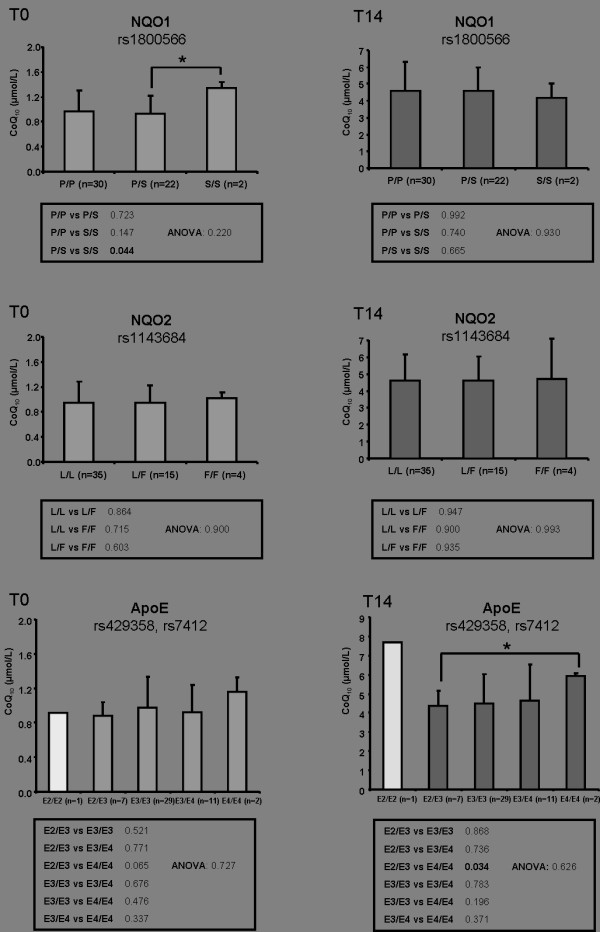
**Effect of NQO1^P187S^, NQO2^L47F ^and apoE genotype distribution on CoQ_10 _plasma levels**. CoQ_10 _plasma levels before (T_0_) and after (T_14_) ubiquinol supplementation (150 mg/day) in humans are shown. Values are mean ± SD and n numbers (genotype distribution) are given in brackets. Differences between two genotype groups were examined using Student *t*-test (*p ≤ 0.05) and between all genotypes using "test for linear trend" (ANOVA).

The selected SNPs were genotyped in 54 healthy male volunteers. The obtained genotype distribution (Figure 1 and 2) were in accordance to the HapMap data: Genotype distribution of the CoQ3^G272S ^polymorphism revealed 38 homozygous for G/G (73%), 13 heterozygous for G/S (25%) and 1 homozygous for S/S (2%), while 1 sample failed genotyping. Analysis of the CoQ6^M406V ^genotype showed 19 homozygous for M/M (36%), 24 heterozygous for M/V (44%) and 11 homozygous for V/V (20%). Genotyping of CoQ7^M103T ^polymorphism revealed 25 M/M (48%), 17 M/T (33%) and 10 T/T (19%) carriers. Two samples failed genotyping. Concerning the distribution of the NQO1^P187S ^SNP, 30 persons are carriers of two P/P alleles (56%), 22 persons were heterozygous with one P and one S allele (41%) and two participants were carriers of two S/S alleles (3%). NQO2^L47F ^genotyping displayed 35 participants were homozygous L/L carriers (65%), 15 participants were heterozygous for L/F (28%) and 4 participants were homozygous F/F carriers (7%). The genotype distribution of apoE was as follows: 1 person with E2/E2 genotype (2%), 7 persons with E2/E3 (14%), 29 persons with E3/E3 (58%), 11 persons with E3/E4 (22%) and 2 persons with E4/E4 (4%). For 4 persons, genotyping of one or both SNPs respectively failed. Thus, the Apo E genotype distribution in our cohort of 54 healthy men was comparable with previously published data [29,30].

### Association between genotypes and CoQ_10 _level at baseline T_0 _and after supplementation T_14 _with the reduced form of CoQ_10_

As previously described [18], 54 healthy male volunteers received 150 mg of the reduced form of CoQ_10 _daily in form of three capsules with each principal meal for 14 days. This supplementation led to a significant 4-fold increase in total CoQ_10 _plasma levels at T_14 _(4.60 ± 1.55 μmol/L) compared to T_0 _(0.96 ± 0.31 μmol/L) [18]. As shown in Figure 1 and 2, SNPs determined in the CoQ7 and NQO2 genes were not associated with total CoQ_10 _levels. Trend analysis (ANOVA) over all genotype variants of CoQ7^M103T ^and NQO2^L47F ^revealed p values >0.05 and were therefore considered as not significant.

### CoQ3^G272S^

The COQ3 gene encodes an O-methyltransferase required for two steps in the biosynthetic pathway of CoQ_10 _[31]. Analysing CoQ3 rs6925344 SNP in association to plasma CoQ_10 _levels at T_0_, no significant differences between genotypes could be revealed. Yet at T_14_, G/S carriers in CoQ3^G272S ^genotype had a higher total CoQ_10 _content (5.30 ± 0.96 μmol/L) after supplementation compared to G/G carriers (4.42 ± 1.67 μmol/L) with borderline significance (p = 0.082, *t*-test).

### CoQ6^M406V^

CoQ6 is mapped to human chromosome 14q24.3 and encodes a monooygenase, which is required in CoQ_10 _biosynthesis for incorporation of oxygen to the benzoquinone ring [32]. CoQ_10 _plasma levels were not significantly changed within genotype distribution of CoQ6 rs8500 SNP before (T_0_) and after (T_14_) supplementation. However, considering total CoQ_10 _distribution at T_0 _in a chi-square cross tabulation as a function of CoQ6 rs8500 genotype (Table 2) a person chi-square χ^2 ^value of p = 0.081 was evident, which again can be considered as marginal significant. Therefore a power calculation for CoQ6 genotype rs8500 was conducted using GPower program (Version 3.1). This disclosed a total of 898 individuals are required to receive 95% power.

**Table 2:** Total CoQ_10 _distribution in a chi-square crosstabulation as a function of CoQ6^M406V ^genotype (rs8500).

	Pearson Χ^2^		
CoQ6 (rs8500)	< 0.96 (μmol/L)	> 0.96 (μmol/L)	Total
**M/M**	7	12	**19**
**M/V+V/V**	21	13	**34**
**Total**	**28**	**25**	**53**

Person Chi-Square Χ^2^: p = 0.081

Distribution was calculated according to a dominant model. CoQ_10 _mean value of 0.96 μmol/L was used for group classification.

### NQO1^P187S^

It has been shown, that NQO1 can generate and maintain the reduced state of ubiquinones in membrane systems and liposomes, thereby promoting their antioxidant function [33,34]. NQO1^P187S ^SNP was associated with CoQ_10 _levels at T_0 _(P/S versus S/S, p = 0.044). Thus, this pilot study indicates that Pro187Ser SNP in NQO1 gene could participate in abnormal CoQ_10 _metabolism. SNP prediction of functional effects of human nsSNPs with structure homology-based method (PolyPhen) revealed a possibly damaging effect of NQO1^P187S ^SNP with a score of 0.215. However, genotype distribution of the S/S genotype was low (n = 2), which reflects the ethnic variation of this polymorphism with the highest prevalence of the S allele in East Asian populations (e.g. 22% prevalence in Chinese populations) and the lowest prevalence in Caucasians (4%) [35]. Furthermore Han et al [36] found a significant association of this SNP with carotid artery plaques in type 2 diabetic patients in east Asian populations. As this genetic variation may play a more significant role in an East Asian rather than in a Caucasian population, evaluation of the Pro187Ser SNP in association with CoQ_10 _metabolism in an East Asian population may be preferable.

### apoE

Apolipoprotein E (apoE) is a polymorphic multifunctional protein with three common isoforms in humans (E2, E3 and E4). Presence of the apoE4 allele is associated with a 40-50% higher risk of cardiovascular disease [37]. There is increasing evidence demonstrating that the apoE4 allele may be associated with elevated oxidative stress and chronic inflammation [38]. Thus apoE was considered as a candidate gene explaining variance in CoQ_10 _status. At T_0_, total CoQ_10 _levels were higher in E4/E4 carriers as compared to all other genotype groups, however p values did not reached significance (p = 0.065, E2/E3 vs E4/E4, Figure 2). These results confirm the results found by Battino et al [29] in a cohort of 106 healthy blood donors. Interestingly, in our study total CoQ_10 _levels increased significantly (p = 0.034) in E4/E4 carriers after supplementation (T_14_), which has to the best of our knowledge not been shown so far. Thus, E4/E4 carriers may be more responsive towards a dietary CoQ_10 _supplementation than non E2/E3 carriers. The underlying physiological and/or molecular mechanisms for this finding still need to be elucidated.

## Conclusions

Taken together, our pilot study with 54 volunteers provides evidence that NQO1^P187S ^and apoE polymorphisms may influence CoQ_10 _status in humans. According to our results and power calculation, larger cohorts are needed in further studies to determine the association between single nucleotide polymorphisms in genes encoding proteins of CoQ_10 _biosynthesis, reduction and metabolism and CoQ_10 _status.

## Competing interests

The authors declare that they have no competing interests.

## Authors' contributions

AF analysed the data and wrote the manuscript. CS participated in the design of the study, acquired and analysed the data. GR participated in the design of the study and critically revised the manuscript. PN and TM carried out the CoQ_10 _measurements. FD was responsible for the concept and design of the study and the writing of the paper. All authors read and approved the final manuscript.
